# Occupational asthma from exposure to rye flour in a Japanese baker

**DOI:** 10.1002/rcr2.63

**Published:** 2014-07-14

**Authors:** Chiyako Oshikata, Naomi Tsurikisawa, Akemi Saito, Hiroshi Yasueda, Kazuo Akiyama

**Affiliations:** 1 Department of Allergy and Respirology, National Hospital Organization Sagamihara National HospitalSagamihara, Japan; 2 Clinical Research Center for Allergy and Rheumatology, National Hospital Organization Sagamihara National HospitalSagamihara, Japan

**Keywords:** Baker’s asthma, bronchial provocation test, occupational asthma, rye flour, wheat flour

## Abstract

Three years after beginning employment at a bakery, a 32-year-old Japanese man began experiencing acute asthma exacerbations after exposure to rye flour. Antigen-specific serum IgE antibodies were detected to the albumin and globulin, gliadin, prolamin, and glutenin protein fractions of rye flour purified from the crude antigen, but only to the albumin and globulin fraction of wheat flour. The histamine concentration producing one-half maximal effect was lower for all four rye flour fractions than for the wheat flour fractions. After inhalation of the albumin and globulin fraction of rye flour, forced expiratory volume in 1 sec decreased to 77.7% of that pre-provocation. To our knowledge, this is the first report of baker’s asthma due to rye flour in Japan.

## Introduction

Baker’s asthma is caused by inhalation of cereal flours and is a common occupational allergic disease in Europe and the United States, where bread is a staple food [1], [2]. Wheat flour-induced asthma has been reported in Japan since the 1980s [3], but until now there have been no reports of rye flour-induced asthma not only in Japan but also in other Asian countries. Here, we present the first report of baker’s asthma caused by exposure to rye flour in a Japanese baker.

## Case Report

A 32-year-old Japanese male ex-smoker (Brinkman index: 390) presented with a history of allergic seasonal rhinoconjunctivitis since the age of 20, but with no history of asthma, although his younger brother did have bronchial asthma. The patient had worked as a baker since the age of 25. Since the age of 27, he had experienced rhinoconjunctivitis exacerbations while handling rye flour but not wheat flour. At the age of 28, he experienced the first episode of chest tightness, cough, and wheezing while handling rye flour; the episode persisted until the next day. At age 32, 30 min after eating 200 g of bread that included 50% rye flour, the patient experienced anaphylactic symptoms, including upper body urticaria, facial swelling, angioedema of the eyelids, and dyspnea, due to narrowing of the respiratory tract.

We purified crude antigen extracted from wheat or rye flour into four protein fractions – albumin and globulin, gliadin, prolamin, and glutenin – as described [4]. We performed a skin prick test and measured antigen-specific serum IgE levels, as described [5]. We also performed histamine release testing by using a Histamine ELISA Kit (Medical & Biological Laboratories Co., Ltd., Nagano, Japan). Skin prick testing was positive (data not shown) and antigen-specific IgE antibodies (Table 1) were detected in all four fractions of rye flour, but only to the albumin and globulin fraction of wheat flour. The histamine concentration producing one-half maximal effect was lower for the four rye flour fractions than for the wheat flour fractions (Table 1).

**Table 1:** Result of antigen-specific IgE and histamine release tests

	Specific IgE antibody (PRU/mL)				EC_50_ (ng/mL)			
	Albumins + Globulins	Prolamin	Gliadins	Glutenins	Albumins + Globulins	Prolamin	Gliadins	Glutenins
Wheat	9.0	<0.35	<0.35	<0.35	250	22	45	17
Rye	19.7	1.17	0.83	1.01	100	3.7	5.5	7.4

EC_50_, histamine concentration producing one-half maximal effect; PRU, protein reference units.

**Figure 1:**
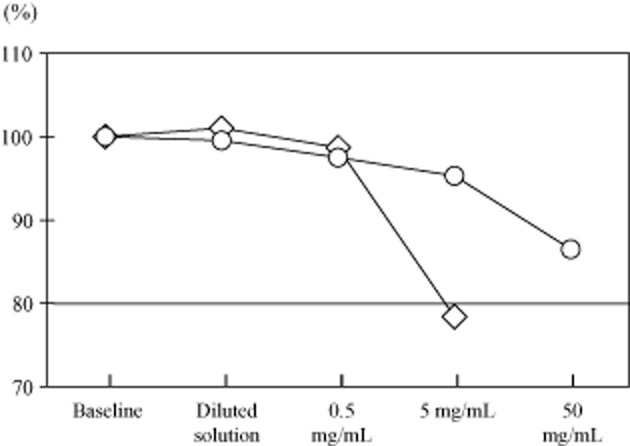
Bronchial provocation testing with the albumin and globulin protein fraction of rye or wheat flour. The patient was exposed to the indicated doses of the albumin and globulin protein fraction of wheat flour (circles) or rye flour (diamonds), and the change in forced expiratory volume in 1 sec (FEV1) from baseline (100%) was recorded. A decrease of more than 20% (horizontal line) from the baseline was defined as a positive reaction to the provocation protein fraction.

There were no abnormal chest radiograph findings. The patient had airway obstruction. His spirometry parameters when not experiencing acute asthma exacerbation were: forced vital capacity (FVC), 4.76 L; percent-predicted FVC, 115.0%; forced expiratory volume in 1 sec, 3.87 L; percent forced expiratory volume in 1 sec, 100.3%; maximum expiratory flow rate at 50% FVC, 3.86 L/sec; percent maximum expiratory flow rate at 50% FVC, 66.0%; maximum expiratory flow rate at 25% FVC, 1.48 L/sec; and percent maximum expiratory flow rate at 25% FVC, 47.9% (Table 2). Ten minutes after antigen-specific provocation with 5 mg/mL of the albumin and globulin fraction of rye flour, the patient developed wheezing and chest tightness; his forced expiratory volume in 1 sec decreased to 77.7% of that pre-provocation (Fig. 1). However, after provocation with 50 mg/mL of the albumin and globulin fraction of wheat flour, lung function did not decrease and no other symptoms were observed. The patient was prescribed fluticasone plus salmeterol (250 + 50 μg) twice daily and 10 mg of a leukotriene receptor antagonist once a day. He continued working at the bakery without further exacerbations by baking only bread made with wheat flour, wearing a mask while working, and avoiding all exposure to rye flour.

**Table 2:** Lung function at first hospital visit

	FEV1	FVC	FEV1/FVC (%)	V50	V25
Littler (L)	3.87	4.76	81.3	3.86	1.48
% predicted	100.3	115.0	93.2	66.0	47.9

FEV1, forced expiratory volume in 1 sec; FVC, forced vital capacity; V25, maximum expiratory flow rate at 25% FVC; V50, maximum expiratory flow rate at 50% FVC.

## Discussion

The incidence of cereal-induced occupational asthma is 0.081/1000 person-years in the UK and 3.4/1000 person-years in Norway [1]. Homology among inhibitor subunits of wheat, rye, and barley partially accounts for the cross-reactivity seen between flours of these cereals. In most patients with baker’s asthma, bronchial asthma is induced by exposure to either wheat or rye flours; however, some patients are allergic to both wheat and rye but the provocation test is only positive for rye [1]. Our patient had serum IgE antibodies to the gliadin, glutenin, and prolamin protein fractions of rye flour, but not to those of wheat flour. Therefore, he had an independent allergy to rye flour. Wheat and rye flour proteins can be classified as either salt soluble (albumins and glutenins) or salt insoluble (prolamins, which include gliadins and glutenins). Salt-soluble proteins are largely associated with baker’s asthma, whereas salt-insoluble proteins are largely associated with wheat-dependent, exercise-induced anaphylaxis; however, patients with food allergies produce IgE to both protein types [1]. We purified crude antigen derived from wheat and rye flour samples provided by the patient into four protein fractions. The albumin and globulin fraction accounted for 77.6% of the crude antigen in rye flour, but only 30.5% of that in wheat flour. Our patient had never experienced asthma symptoms after handling wheat flour. We were unable to perform antigen-specific provocation testing with the gliadin, prolamin, or glutenin protein fractions because they are insoluble. However, both antigen-specific serum IgE and histamine release testing for the gliadin, prolamin, and glutenin fractions of rye flour were positive. Therefore, it was possible that this patient experienced acute asthma exacerbation after exposure not only to the albumin and globulin fraction of rye flour, but also to the gliadin, prolamin, or glutenin fractions.

Recently, the demand for bread made with rye flour has increased in Japan because of the diversification of eating habits and greater concern for a healthy lifestyle. This is the first report of baker’s asthma caused by exposure to rye flour in a Japanese patient. This report will contribute to progress in the diagnosis and management of occupational asthma.
